# Real-World Utilization of Corticosteroids in Severe Alcoholic Hepatitis: Eligibility, Response, and Outcomes

**DOI:** 10.3390/medicina60020311

**Published:** 2024-02-11

**Authors:** Ana-Maria Singeap, Horia Minea, Oana Petrea, Madalina-Andreea Robea, Ioana-Miruna Balmuș, Raluca Duta, Ovidiu-Dumitru Ilie, Carmen Diana Cimpoesu, Carol Stanciu, Anca Trifan

**Affiliations:** 1 Department of Gastroenterology, Faculty of Medicine, University of Medicine and Pharmacy “Grigore T. Popa”, 700115 Iasi, Romania; anamaria.singeap@yahoo.com (A.-M.S.); stanciucarol@yahoo.com (C.S.); ancatrifan@yahoo.com (A.T.); 2 Institute of Gastroenterology and Hepatology, “St. Spiridon” University Hospital, 700111 Iasi, Romania; 3 CENEMED Platform for Interdisciplinary Research, University of Medicine and Pharmacy “Grigore T. Popa”, 700115 Iasi, Romania; madalina.robea11@gmail.com (M.-A.R.); balmus.ioanamiruna@yahoo.com (I.-M.B.); duta.raluca112@gmail.com (R.D.); ovidiuilie90@yahoo.com (O.-D.I.); carmen.cimpoesu@umfiasi.ro (C.D.C.); 4 Department of Exact Sciences and Natural Sciences, Institute of Interdisciplinary Research, “Alexandru Ioan Cuza” University of Iasi, 700057 Iasi, Romania; 5 Department of Mother and Child, Faculty of Medicine, University of Medicine and Pharmacy “Grigore T. Popa”, University Street, No. 16, 700115 Iasi, Romania; 6 Department of Emergency Medicine, “St. Spiridon” University Hospital, 700111 Iasi, Romania; 7 Faculty of Medicine, University of Medicine and Pharmacy “Grigore T. Popa”, Iasi, Blvd. Independentei 1, 700111 Iasi, Romania; 8 Centre of Biomedical Research, Romanian Academy, Carol I Avenue, No. 8, 700506 Iasi, Romania

**Keywords:** severe alcoholic hepatitis, corticosteroids, mortality rate, prognostic factors

## Abstract

*Background and Objectives:* Alcoholic hepatitis (AH) poses a medical challenge, causing moderately severe to life-threatening episodes with high short- and long-term mortality. This study aimed to explore real-world corticosteroid utilization in severe AH, response predictors, and patient outcomes. *Materials and Methods:* We conducted a retrospective study on patients admitted for severe AH, defined as a Maddrey Discriminant Function score equal to or above 32, at a tertiary care center. We reviewed patients’ medical observation charts to identify corticosteroid prescriptions, reasons for ineligibility, and response rates. Responders were defined based on the Lille score, and predictors of non-response were identified. Short-term (one-month) and long-term (one-year) mortality rates were calculated according to treatment and response. *Results:* Out of 310 patients enrolled with severe AH, 59% received corticosteroids, achieving a response rate of 75.4%. The reasons for not administering corticosteroids were as follows: uncontrolled infections (27.6%), renal dysfunction (20.4%), gastrointestinal bleeding (18.9%), acute pancreatitis (7.1%), uncontrolled diabetes (3.1%), and other or unknown causes (22.8%). The overall 1-month mortality rate was 12.2%, higher in non-responders (35.3%) and patients who did not receive corticosteroids (13.4%) compared to responders (3.6%). The overall 1-year mortality rate was 62.5%, similar between patients who did not receive corticosteroids (78.7%) and non-responders (77.7%) and higher compared to responders (42.8%). Predictive factors for non-response included older age (OR = 1.05, 95%CI: 1.01–1.08), concomitant cirrhosis (OR= 2.11, 95% CI: 1.064–4.20), MELD scores exceeding 30 (OR = 2.42, 95% CI: 1.21–4.80), severe hypoalbuminemia (OR = 2.46, 95%CI: 1.12–5.37), and increased serum creatinine (OR = 1.5, 95% CI: 1.1–2.03). Among the prognostic scores, MELD 3.0 score exhibited superior efficacy for short-term (AUC = 0.734, 95% CI 0.656–0.811) and long-term mortality (AUC = 0.777, 95% CI: 0.724–0.830) compared to alternative scoring systems. *Conclusions:* Low eligibility rate and poor prognosis underscore the need for effective therapies. Our findings contribute to refining risk stratification and early prediction of non-response, aiding clinicians in identifying more beneficial therapies.

## 1. Introduction

Alcoholic hepatitis (AH), one of the most serious consequences of alcohol abuse, stands as a critical medical concern. Acute liver injury, induced by excessive alcohol consumption, poses an exceptional challenge, given its potential for life-threatening outcomes. The overall mortality rate for AH episodes is approximately 30–40% at 90 days [1]. While mild-to-moderate cases of AH may benefit from conventional measures, primarily involving alcohol cessation and nutritional support, severe AH requires intensive care, close monitoring, complex support interventions, and specific treatments. A widely employed clinical tool for assessing the severity of AH is the Maddrey Discriminant Function (MDF) score, calculated using a formula that considers the patient’s prothrombin time and serum bilirubin level [2]. Severe AH is typically defined by a score of 32 or higher, and this is associated with an increased short-term mortality risk, reaching up to a 50% mortality rate within 28 days [2,3,4]. This score serves to identify individuals at higher risk of poor outcomes who, at the same time, might benefit from steroid administration. While a large randomized clinical trial (STOPAH) did not demonstrate a significant decrease in 30-day mortality among patients with severe AH treated with corticosteroids [5], other studies have shown advantages of corticosteroid treatment, particularly in terms of short-term survival [6,7]. Despite some conflicting evidence, clinical guidelines currently recommend corticosteroids as the first-line pharmacological therapy for severe AH [8,9]. In addition to MDF, other scores have demonstrated their correlation with mortality and can be used in clinical practice as prognostic models. These include a MELD (Model for End-stage Liver Disease) score over 20, an ABIC score (based on age, serum bilirubin, international normalized ratio, and serum creatinine) category C, or a Glasgow AH score of 9 or higher [10].

Despite the superior accuracy of the MELD-Na score compared to MELD and MDF, there is the possibility of underestimating the severity of this disease in women or malnourished patients [11].

To eliminate these disadvantages and obtain an improved prediction of mortality for decompensated liver cirrhosis, the MELD 3.0 score was defined, in which the female gender and serum albumin concentration were included as variables [12].

Corticosteroids, administered as a short course of prednisolone (40 mg/day for 28 days) or an equivalent, currently serve as the cornerstone of treatment for severe AH. Traditionally, on day 7 following the initiation of treatment, the Lille score must be calculated to determine if the patient is responding to therapy and whether it should be continued or discontinued. A Lille score above 0.45 indicates a lack of response, and continuing corticosteroids will not yield benefits and may even be detrimental, whereas a Lille score below 0.45 signifies a positive response, justifying the continuation of treatment. A Lille score above 0.45 is associated with 75% mortality rate at 6 months [13]. However, for patients who initially respond, the long-term survival benefits remain uncertain [14,15]. When corticosteroids are contraindicated, pentoxifylline has been proposed as an alternative with short-term survival benefits, although long-term survival improvement has not been definitively established [5]. The role of liver transplantation in the management of severe AH is controversial, with questions raised about its benefits in terms of relapse risk, organ scarcity, and ethical considerations [16].

Although corticosteroids are considered the first-line treatment for severe AH, they fall short of being the ideal choice due to their side effects and relatively low response rates. Furthermore, various contraindications may prevent their use, and in reality, other factors can reduce eligibility for corticosteroid treatment, which can present a challenging issue. 

Accurate risk stratification and early prediction of non-response are essential for preventing patients from unnecessary risks and for helping clinicians identify alternative therapies or interventions that may offer greater benefits. 

Our objective was to evaluate the real-world utilization of corticosteroids for severe AH, with a focus on the frequency of prescription, reasons for ineligibility, response rates, predictors for non-response, and patient outcomes, specifically in terms of short- and long-term mortality. Furthermore, our secondary objective aimed to assess the current scoring systems’ effectiveness in predicting mortality at one month and one year.

## 2. Materials and Methods

### 2.1. Patient Selection

We conducted a retrospective study on patients admitted for AH at the Institute of Gastroenterology and Hepatology in Iasi, a tertiary care center in northeastern Romania, covering the period from January 2019 to August 2022. We reviewed medical observation charts and included in our analysis patients with severe AH, defined as those with a MDF score equal to or above 32. These patients represented the cohort for whom corticosteroid treatment was theoretically indicated. 

### 2.2. Methods

We recorded clinical and laboratory parameters for all enrolled in the study. Corticosteroid administration was evaluated for each patient. The eligibility rate was defined as the proportion of patients who received corticosteroids out of the total number of patients with severe AH. In cases where corticosteroids were not recommended, we determined the specific reason. The reasons for non-prescription were categorized into two groups: contraindications for corticosteroids and other reasons. Contraindications for corticosteroids included conditions such as uncontrolled infections, gastrointestinal bleeding, renal failure, uncontrolled diabetes, acute pancreatitis, and psychosis. Responders to corticosteroid treatment were identified using the Lille score on the seventh day of treatment.

We analyzed clinical and laboratory parameters as predictive factors for non-response to corticosteroids. Short-term (one-month) and long-term (one-year) mortality rates were calculated according to the patient’s specific management (corticosteroids or not) and response status.

MELD, MELD Na, MELD 3.0, MDF, GAHS (Glasgow Alcoholic Hepatitis Score) and ABIC (Age–Bilirubin–International Normalized Ratio–Creatinine) scores were recorded to predict mortality at one month and one year, respectively, being calculated using the following formulas [10,17]: MELD (Model for End-stage Liver Disease) = 9.57 × loge (creatinine) + 3.78 × loge (total bilirubin) + 11.2 × loge (INR) + 6.43; MELD-Na (Model for End-stage Liver Disease sodium)= MELD + 1.32 × (137 − Na) − [0.033 × MELD × (137 − Na)]; MELD 3.0 = 1.33 (if female) + 4.56 × log_e_(bilirubin) + 0.82 × (137 − Na) − 0.24 × (137 − Na) × log_e_(bilirubin) + 9.09 × log_e_(INR) + 11.14 × log_e_(creatinine) + 1.85 × (3.5 − albumin) − 1.83 × (3.5 − albumin) × log_e_(creatinine) + 6; Maddrey DFI = 4.6 × (PTsec-control PTsec) + serum total bilirubin in mg/dL; and ABIC score = (age × 0.1) + (serum bilirubin × 0.08) + (serum creatinine × 0.3) + (INR × 0.8). GAHS is calculated using the addition of selected points for the following parameters: age (1 point if age < 50 years or 2 points if ≥50 years), white blood cell count (1 point if <15 × 10^9^/L or 2 points if ≥15 × 10^9^/L), BUN (blood urea nitrogen) (1 point if <5 mmol/L or 2 points if ≥5 mmol/L), bilirubin (1 point if <125 μmol/L, 2 points if 125–250 μmol/L or 3 points if ≥250 μmol/L), and PT ratio (prothrombin time) (1 point if <1.5, 2 points if 1.5–2.0, or 3 points if >2.0).

### 2.3. Data Analysis

Statistical analyses were conducted using IBM SPSS software, version 26.0 (IBM SPSS Inc., Chicago, IL, USA). OR (odds ratio) was used to identify risk factors for non-response to corticotherapy. Quantitative variables were presented as medians and IQRs (interquartile ranges), while qualitative variables were expressed as numbers and frequencies. The efficacy of the predictive scores was assessed using ROC (receiver operating characteristic) curves. The *p*-value was established to be equal to or less than 0.05 for both statistical tests performed.

## 3. Results

### 3.1. Baseline Patient Characteristics

A total of 310 patients, comprising 199 men and 111 women, were hospitalized for severe AH during the study period, with ages ranging from 27 to 78 years.

The baseline demographic, clinical, and biological characteristics of the patients are presented in Table 1. Approximately three-quarters of the patients had a prior diagnosis of liver cirrhosis. Among cirrhotic patients, the majority presented with ascites (197 patients, 85%), while in patients without a previous cirrhosis diagnosis, ascites accompanied the clinical picture in 11 cases (27.5%). Overt hepatic encephalopathy was observed in about half of all patients (51%).

### 3.2. Corticosteroid Eligibility

Corticosteroids were initiated in 183 patients, corresponding to an eligibility rate of 59% (Table 2).

For the remaining 127 (41%) patients, the reasons for the lack of administration were as follows: uncontrolled infections in 35 cases (27.5%), including bacterial respiratory infections (including *Mycobacterium tuberculosis*) (27%), spontaneous bacterial peritonitis (32%), urosepsis (28%), cellulitis (10%), or replicative viral hepatitis (3%); renal dysfunction in 26 cases (20.4%); gastrointestinal bleeding in 24 cases (18.8%); acute pancreatitis in 9 cases (7.1%); uncontrolled diabetes in 4 cases (3.1%); and other reasons in 29 patients (22.8%). There were no cases of psychosis. Due to the retrospective nature of this study, we could not identify the exact reason for not administering corticosteroids in these 29 patients. However, 22 patients had either an MDF score above 90 or a MELD score of more than 52, which could have influenced the clinical decision. In the case of four patients, their condition rapidly deteriorated (within 36 h after presentation), and there was insufficient time to complete infectious screening and initiate corticosteroid treatment.

### 3.3. Outcomes: Response Rate, Short-Term and Long-Term Mortality

Out of all the 183 patients who received corticosteroids, 138 (75,4%) were identified as responders, with a 7-day Lille score of less than 0.45. Within the first month after the baseline, 38 patients died, resulting in a 12.2% short-term mortality rate. According to the treatment strategy, short-term mortality rates were as follows: 3.6% in corticosteroid responders, 35.5% in corticosteroid non-responders, and 13.39% in patients who did not receive corticosteroids. For the first year of follow-up, another 156 patients died, resulting in a mortality rate among short-term survivors of 50.3%, and a total long-term mortality rate of 62.5%. According to treatment, the long-term mortality rates were 42.7% for corticosteroid responders, 77.7% for corticosteroid non-responders, and 78.7% for patients who did not receive corticosteroid treatment (Table 3).

The mortality risk was assessed based on corticosteroid administration and response. The results indicated that, both in the short and long term, patients who did not respond and patients who did not receive corticosteroids had significantly higher mortality rates compared to responders (Table 4).

### 3.4. Predictive Factors for Non-Response to Corticosteroids

We assessed correlations between clinical and biological parameters and response to corticosteroids (Table 5).

According to clinical features, we found that higher age (OR = 1.05, 95% CI: 1.01–1.08) and the presence of preexisting cirrhosis (OR = 2.11, 95% CI: 1.06– 4.20) correlate with the lack of response to corticosteroid treatment. Simultaneously, elevated serum creatinine (OR = 1.50, 95% CI: 1.10–2.03) was associated with non-response. Furthermore, the cut-off value of 2.8 mg/dL used to define severe hypoalbuminemia correlates with a lack of response (OR = 2.46, 95% CI: 1.12–5.37). In terms of MDF score, higher values were significantly linked to a higher likelihood of non-response, but without identifying a specific cut-off value correlated to the lack of response. We found no correlations between gender, the presence of ascites, and other biological parameters, including platelet count, hemoglobin levels, prothrombin time and INR, serum total bilirubin, and sodium levels. MELD values were higher in non-responders. Additionally, when a cut-off value set at over 30 was applied for the MELD score, a correlation with a lack of response was noted (OR = 2.42, 95% CI: 1.21–4.80).

### 3.5. Assessing the Risk of Mortality through Predictive Scores

**Figure 1:**
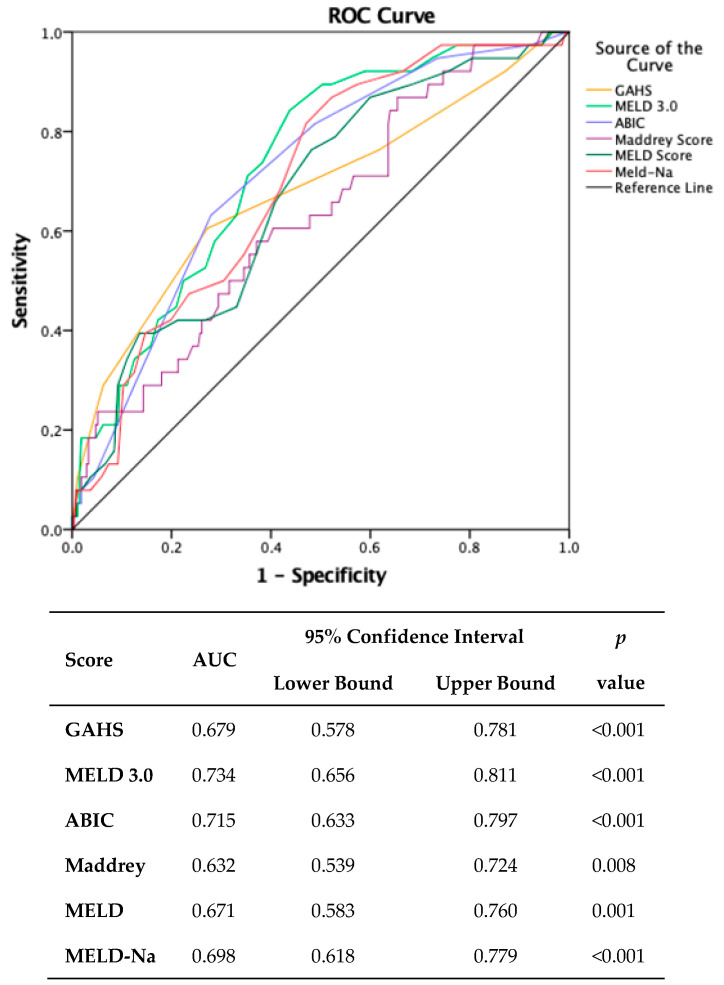
Comparison of scores predicting one-month mortality. (MDF: Maddrey Discriminant Function; MELD: Model for End-stage Liver Disease; MELD-Na: Model for End-stage Liver Disease sodium; MELD 3.0: Model for End-stage Liver Disease 3.0; GAHS: Glasgow Alcoholic Hepatitis Score; ABIC: Age–Bilirubin–INR–Creatinine.).

To proactively assess adverse outcomes, six scores were conducted upon admission. Receiver operating characteristic (ROC) curve analysis revealed that MELD 3.0 scores exhibited superior predictive efficacy for one-month mortality (AUC = 0.734, 95% CI 0.656–0.811, *p* < 0.001) compared to alternative scoring systems. Notably, a MELD 3.0 score exceeding 20 demonstrated a sensitivity of 92.1% and a specificity of 37% (Figure 1).

Concerning one-year outcomes, the findings indicated that the same scoring system exhibited the highest efficacy (AUC = 0.777, 95% CI: 0.724–0.830, *p* < 0.001).

**Figure 2:**
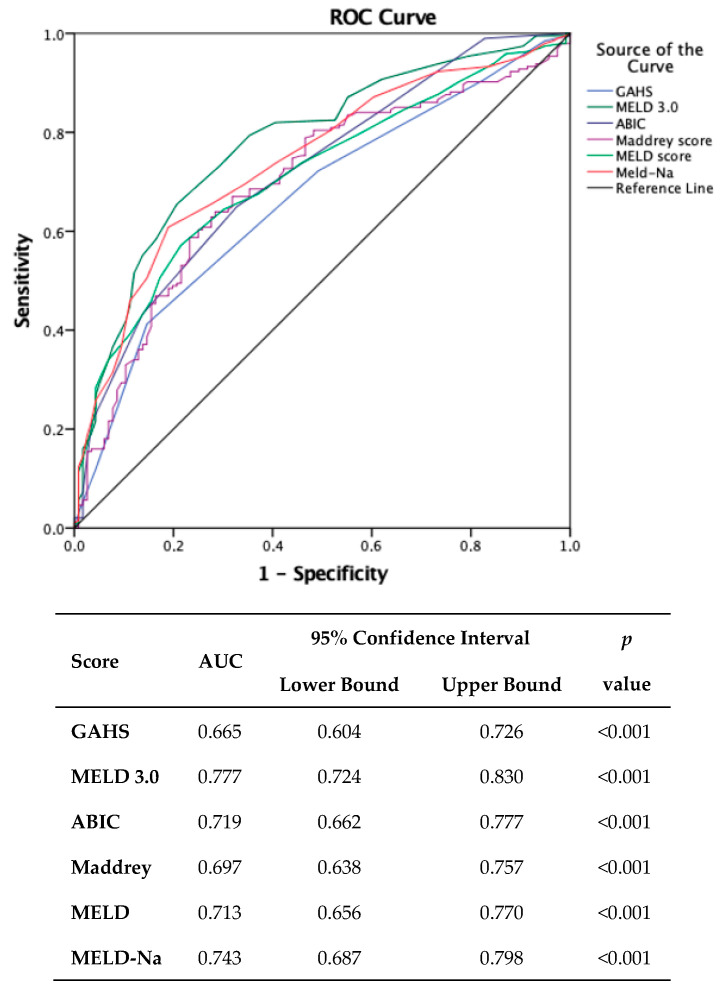
Comparison of scores in predicting one-year mortality. (MDF: Maddrey Discriminant Function; MELD: Model for End-stage Liver Disease; MELD-Na: Model for End-stage Liver Disease sodium; MELD 3.0: Model for End-stage Liver Disease 3.0; GAHS: Glasgow Alcoholic Hepatitis Score; ABIC: Age–Bilirubin–INR–Creatinine.).

Establishing the same threshold at admission, this score demonstrated a sensitivity of 91% and a specificity of 48% (Figure 2).

## 4. Discussion

Severe AH remains a condition characterized by high mortality rates and limited treatment options.

Its unfavorable outcomes are closely tied to intense liver inflammation resulting from hepatocyte necrosis, which exacerbates liver injury and impedes the process of tissue repair and regeneration [18]. Severe AH often leads to systemic inflammatory response syndrome and multi-organ failure, with short-term mortality rates reaching 40% [19]. Corticosteroids are employed in the treatment of severe AH due to their anti-inflammatory properties, although the results are not uniform. The rationale behind their use lies in their ability to suppress the inflammatory response and mitigate immune-mediated liver damage. They achieve this by reducing the infiltration of polymorphonuclear neutrophils into the liver and rebalancing cytokine production [20]. Pro-inflammatory cytokines like tumor necrosis factor-alpha, intercellular adhesion molecule 1, interleukin-6, and interleukin-8 are decreased, while anti-inflammatory cytokines, such as interleukin-10, are increased [21]. Early studies indicated a short-term survival improvement in patients with severe AH when treated with corticosteroids [6,22,23]. However, post-treatment infections were found to lead to an increased mortality rate [24]. The STOPAH trial, a large multi-center study involving more than 1000 patients, randomly assigned patients to evaluate the effects of prednisolone and pentoxifylline versus placebo [5]. The results indicated that prednisolone was associated with a reduction in 28-day mortality, although it did not reach significance. Moreover, no improvement was observed in outcomes at ninety days or one year, and pentoxifylline did not demonstrate a survival benefit [5]. The response rate to corticosteroids was reported as being in the range of 50–60% [5].

While the results in the literature have been inconsistent, corticosteroids remain the primary treatment for severe AH at present. Nevertheless, the presence of contraindications and the potential for complications and adverse events limit the administration of corticosteroids in many cases, necessitating careful consideration before their use.

In our study, we noted an eligibility rate for corticosteroids of 59%, with a favorable response rate of 75.4%. A recent study conducted by Shasthry et al. revealed that among 430 patients hospitalized with severe AH, only 132 individuals (26.8%) met the eligibility criteria. Among this cohort, 99 patients (75%) responded favorably to corticosteroids [18]. However, in this analysis, a considerable number of patients initially considered for corticosteroids were excluded due to factors such as death (86 out of 430), discharge against medical advice (64 individuals), low MDF score, or spontaneous improvement. If we hypothetically recalculated the respective eligibility rate by excluding these cases, it would reach just over 50%, aligning with the eligibility rate observed in our study. Another retrospective study found a higher eligibility rate, with 74% of the 103 enrolled patients receiving corticosteroids, while 26% had contraindications [25].

Common contraindications for corticosteroid treatment in cases of severe AH include active infections, renal injury, gastrointestinal bleeding, uncontrolled diabetes, acute pancreatitis, and psychosis. Infections are the most prevalent contraindication for corticosteroids, as approximately one quarter of patients with severe AH may have concurrent infections upon admission [24]. While recent data suggest that infections might not necessarily be considered an absolute contraindication, as prior recommendations suggested [24,26], it is essential to achieve resolution or, at the very least, clear control of the infection before initiating corticosteroid treatment. In our study, we identified infections as the most prevalent contraindication for corticosteroid therapy, accounting for 27.5% of the reasons for non-eligibility. The second most common reason for not administering corticosteroids was impaired renal function, representing 20.4% of the causes for non-eligibility. Hepato-renal syndrome has been reported to occur in approximately 10% of cirrhotic patients with ascites [27]. In our study, three-quarters of the patients were classified as having cirrhosis, with the majority in a decompensated stage. At the same time, multiple potential concomitant causes, such as prerenal failure, infections, and nephrotoxic drugs, whether with or without underlying chronic kidney disease, help explain the relatively high number of patients with compromised renal function.

Gastrointestinal bleeding ranked as the third most frequent contraindication for corticosteroids. Given that severe and excessive alcohol consumption is a precipitating factor for upper gastrointestinal hemorrhage, our analysis revealed a relatively high number of patients presenting with concomitant bleeding, comprising 18.8% of the factors leading for non-eligibility.

Regarding the 29 patients with severe AH who did not have clear contraindications for steroid treatment but were not administered steroids (representing 22.8% of the non-eligible group), the retrospective nature of our study prevented us from precisely identifying the specific reasons. Nevertheless, several explanations may account for this.

Firstly, in many studies, the MELD score has proven to be a valuable clinical prediction tool. It demonstrates a strong correlation with 30-day and 90-day mortality, with statistical advantages over MDF [28]. Furthermore, a recent large retrospective international multi-center study, encompassing over 3000 patients with AH, revealed that the maximal benefit from corticosteroid treatment was observed in patients with MELD scores between 25 and 39. However, in patients with the most severe liver disease, i.e., those with a MELD score above 51, the treatment benefit was lost [29]. At the same time, there have been studies examining the concept of an MDF futility score. An earlier study found that the mortality rate reaches nearly 100% when the MDF score surpasses 54, even when steroids are administered [30]. However, more recently, another study showed similar mortality rates, of 21% and 23%, in patients with MDF scores exceeding this value, both in those who received corticosteroid treatment and those who did not, respectively, suggesting that corticosteroid treatment does not enhance survival in patients with MDF scores greater than 54 [31]. Thus, it appears there is a critical window of opportunity for corticosteroid intervention, beyond which no significant benefit may be expected. This might explain the rationale for non-eligibility in some patients in our study. However, a clear definition for patients who are “too ill for corticosteroids” has not yet been established, and future studies are needed in this regard. In our analysis, elevated MDF scores demonstrated a significant association with an increased probability of non-response. At the same time, MELD values were higher in non-responders, and a correlation was observed with the lack of response. Additionally, a specific cut-off value predictive of non-response was identified for MELD score, set at over 30. Secondly, no histological diagnosis was obtained for the patients in our study; instead, the diagnosis of AH was based solely on clinical criteria. However, transjugular liver biopsy was not a prerequisite in most studies [30], since strict clinical criteria ensure a high level of diagnostic accuracy [32]. Nevertheless, the possibility of an infection-related decompensation mimicking acute severe AH could be a reason for withholding corticosteroid treatment in some patients, whether or not active infection was confirmed.

The response rate of 75.4% seems to be one of the highest reported so far. Louvet et al. found a 62% response rate in their prospective inclusion of 118 patients with severe AH during their validation of the Lille model cohort [33]. A retrospective analysis of a cohort of patients with severe AH treated with corticosteroids showed a rate of response of 54%, as determined by the Lille score, calculated after seven days of treatment [34]. A lower response rate, of 36%, was found in another smaller retrospective study conducted two years ago by Foncea et al. [25]. The high response rate we observed may be attributed to the strict selection criteria applied for corticosteroid treatment, particularly in terms of excluding any potential contraindications. In our analysis, no patients with current infection or gastrointestinal bleeding were treated with corticosteroids. Moreover, the potential for an infection-induced decompensation resembling acute severe AE might justify refraining from administering corticosteroids in certain patients, regardless of the confirmation of an active infection. In terms of severe AH and gastrointestinal bleeding, a prior retrospective study found that survival was not significantly different between corticosteroid-treated patients with severe AH presenting with or without hemorrhage (74% compared to 70%, respectively), and that patients with bleeding had lower susceptibility to develop infections [35]. These results may serve as proof that patients with severe AH and recent gastrointestinal bleeding should not be excluded from receiving corticosteroids. However, the data available so far lack uniformity, as another retrospective analysis identified gastrointestinal bleeding as an independent risk factor for one-month and three-month mortality [34]. Nevertheless, it is important to note that the authors of that study did not distinguish between gastrointestinal bleeding present on admission and that which occurred during hospitalization [34].

Many studies so far have consistently demonstrated that an early improvement in liver function, evaluated by the Lille score on day 7 of corticosteroid treatment, is strongly correlated with a reduction in short-term mortality [18,34]. In our analysis, the overall short-term mortality rate, assessed at one month, was 12.2%, significantly higher in non-responders (35.5%) and in patients who did not receive corticosteroid treatment (13.3%) compared to responders (3.6%). The one-month mortality rate identified in our analysis was lower than that reported by Mathurin et al. in their meta-analysis of five randomized controlled trials concerning corticosteroid responders (15%) and non-responders (47%) [22]. Additionally, in our analysis, the one-year overall mortality rate was 62.5%, showing comparable rates between non-responders (77.7%) and patients who did not receive corticosteroids (78.7%). Both groups exhibited significantly higher mortality rates compared to patients who responded to corticoid therapy (42.7%). Relative to one-year overall mortality rates reported in previous studies, such as 43% in a prospective study by Dhanda et al. [36] or 56% as reported by Thursz et al. in the multi-center randomized trial STOPAH [5], our rate of 62.5% is higher. This difference is likely attributed to the substantial proportion (three-quarters) of patients with cirrhosis, the majority of them being decompensated (with ascites), as prognosis in this group of patients is typically poor even in the absence of severe acute hepatitis.

Considering the link between non-response and elevated mortality, especially with a documented mortality rate of non-responders as high as 35.5%, surpassing one-third, the designation of predictors becomes both useful and imperative. In our analysis, factors predicting non-response encompassed older age, concomitant cirrhosis, overt hepatic encephalopathy, hypoalbuminemia, higher MELD scores (over 30), and increased serum creatinine. Age and hypoalbuminemia may be linked to non-response through the lens of frailty. The association between preexistent cirrhosis and non-response may be explained by the heightened susceptibility of the liver due to prior damage. In this context, an acute episode of severe AH appears to act as a precipitating event, triggering decompensation and exacerbating the progression of liver failure. Additionally, elevated MELD scores, signifying advanced liver disease, imply a diminished likelihood of a favorable treatment response [37]. Renal dysfunction was identified as a major contraindication for corticosteroids; nevertheless, slightly elevated values above the normal limit were recorded in some patients treated with corticosteroids, and these values were subsequently correlated with a lack of response. All these findings suggest the importance of addressing modifiable factors as a strategic approach to ensure improved outcomes.

The therapeutic options available to severe AH patients who do not respond to steroids are narrowly limited. N-acetylcysteine did not show a benefit compared to steroids, while combination therapy with steroids increased one-month survival but did not influence the six-month survival rate [4,35,38]. Pentoxifylline has been suggested as an alternative for patients with contraindications for corticosteroids [26,39]. However, even if prior results have shown a decrease in fatal hepato-renal syndromes compared to placebo [40], no benefit in terms of survival was noted at one month [5].

Given the high short-term mortality rates and the absence of effective medical treatments, liver transplantation emerges as a salvage option for these patients. It is the best treatment for severe AH, as showed by studies proving that early liver transplantation improves survival in patients with a first episode of severe AH not responding to medical therapy [41,42,43], while survival rates after LT are comparable to those of patients undergoing LT for other indications [44]. However, there is still uncertainty regarding the feasibility of liver transplantation for these patients, due to the limited clinical data available and the presence of ethical concerns [45]. Moreover, as transplant centers typically mandate a minimum six-month period of alcohol abstinence, and there is a well-known organ shortage, the matter of liver transplantation as a last-resort solution in non-responsive patients with severe AH remains controversial. Furthermore, concerns about relapse and the general public perception that alcoholic liver disease is a self-induced, behavior-related condition pose more challenges when contemplating liver transplantation as a treatment option for these patients.

Nevertheless, some progress has been made in recent years for corticosteroid non-responders. Besides appropriate nutrition—for which, more recently, the designated term has been “aggressive immune-nutrition”—other promising modulation strategies have evolved, such as granulocyte colony-stimulating factor administration and fecal microbiota transplantation [46,47]. There are emerging data on plasma exchange as an effective bridge to liver transplantation, but caution is needed because of the risk of infections and hypervolemia [48,49].

Due to the complex mechanisms underlying AH, applying multiple therapeutic strategies might be the key, and further studies are expected to address hepatic inflammation, infection suppression, and liver regeneration [50]. However, until new therapies become standard practice, the identification of predictors of non-response would be highly valuable for implementing an effective tailored treatment strategy.

Regarding the efficacy of the available scores to predict mortality, MELD 3.0 assumes notable significance, especially considering its introduction of dimensions that bear biological and clinical relevance. Furthermore, it proficiently mitigates the gender disparity engendered by the utilization of MELD or MELDNa in the liver allocation process [12].

This assertion finds support in the findings of Diaz et al., who determined that MELD 3.0 scores at admission exhibited enhanced predictive performance for one-month mortality compared to other traditional scoring systems, yielding an AUC of 0.761 (95% CI: 0.732–0.791). Notably, a score exceeding 20 demonstrated a sensitivity of over 90% in predicting 30-day mortality, aligning closely with the outcomes of the present study [17].

## 5. Conclusions

Severe AH remains a condition with a high short- and long-term mortality rate, particularly among non-responders to corticosteroids. The combination of a low eligibility rate and a poor prognosis underscores the pressing need for effective therapies. Accurate patient selection is a key factor, and our study identified older age, concomitant cirrhosis, low serum albumin, higher serum creatinine, and MELD scores exceeding 30 as predictive factors for non-response. These insights highlight the importance of carefully considering patient characteristics in the decision-making process for corticosteroid treatment in severe AH. Addressing modifiable factors in a timely manner could lead to better outcomes. Until novel therapies become available, our findings contribute to optimizing outcomes in this challenging patient population.

## Figures and Tables

**Table 1:** Baseline patient characteristics.

	Overall *n* = 310	No Corticotherapy *n* = 127	Corticotherapy with Response *n* = 138	Corticotherapy without Response *n* = 45
Age, years, median (IQR)	49 (43 to 59)	51 (44 to 61)	47 (38.75 to 54)	49 (45 to 63)
Gender ratio, men/women, *n* (%)	199/111 (64.2/35.8)	83/44 (65.3/34.7)	88/50 (63.80/36.2)	28/17 (62.20/37.80)
Maddrey score, median (IQR)	63.80 (48.23 to 86)	70.70 (55.30 to 90)	57.10 (42.75 to 79.15)	74 (48.50 to 93.75)
MELD score, median (IQR)	29 (25 to 33)	30 (26 to 35)	27 (24 to 31)	29 (25.50 to 34.50)
MELD-NA score, median (IQR)	30 (26 to 34)	31 (27 to 35)	27 (25 to 32)	31 (27.50 to 34)
GAHS score, median (IQR)	9 (8 to 10)	9 (8 to 10)	9 (8 to 10)	9 (8 to 10)
MELD 3.0 score, median (IQR)	28 (21.75 to 35)	31 (25 to 38)	24 (20 to 30)	31 (24.50 to 35.50)
ABIC score, median (IQR)	9 (8 to 10)	9 (8 to 10)	8 (7 to 9)	9 (8 to 10)
Cirrhosis, yes/no, *n* (%)	232/78 (74.83/25.16)	13/114 (10.20/89.80)	95/43 (68.80/31.20)	23/22 (51.10/48.90)
Ascites, present/absent, *n* (%)	213/97 (68.7/31.3)	83/44 (65.50/34.50)	93/45 (67.40/32.60)	37/8 (82.20/17.80)
Hepatic encephalopathy, present/absent, *n* (%)	158/152 (51/49)	72/55 (56.70/43.30)	58/80 (42/58)	28/17 (62.20/37.80)
Platelets, n × 10^9^/L, median (IQR)	126.50 (77 to 188)	126 (78 to 191)	135.50 (77.75 to 188.50)	110 (64.50 to 190)
Hemoglobin value, mg/dL, median (IQR)	11.10 (9.30 to 12.40)	10.80 (8.70 to 12.20)	11.70 (10.30 to 12.50)	10.60 (9.45 to 12.10)
Prothrombin time, s, median (IQR)	22.70 (19.20 to 27.32)	23.70 (20.80 to 28.90)	21.55 (18.20 to 25.60)	23.20 (17.85 to 26.90)
INR, median (IQR)	2.05 (1.76 to 2.48)	2.15 (1.88 to 2.61)	1.69 (1.97 to 2.33)	2.13 (1.68 to 2.53)
Serum albumin, mg/dL, median (IQR)	2.49 (2.15 to 2.86)	2.32 (1.97 to 2.60)	2.67 (2.33 to 3.05)	2.45 (2.17 to 2.76)
Serum total bilirubin, mg/dL, median (IQR)	18.23 (11.10 to 26.05)	14.85 (8.81 to 25.26)	17.99 (12.15 to 26.92)	22.42 (15.77 to 28.50)
Serum creatinine, mg/dL, median (IQR)	0.82 (0.63 to 1.60)	1.02 (0.68 to 2.27)	0.70 (0.55 to 1.01)	0.95 (0.68 to 1.83)
Serum sodium, mmol/L	132 (128 to 136)	131 (126 to 134)	133 (130 to 136)	132(129 to 135)

MDF: Maddrey Discriminant Function; MELD: Model for End-stage Liver Disease; MELD-Na: Model for End-stage Liver Disease-sodium; MELD 3.0: Model for End-stage Liver Disease3.0; GAHS: Glasgow Alcoholic Hepatitis Score; ABIC: Age–Bilirubin–INR–creatinine.

**Table 2:** Eligibility for corticosteroids.

	Total Number of Patients with Severe AH n = 310
Corticosteroid treatment	
Yes, *n* (%)	**183 (59%)**
No, *n* (%)	**127 (41%)**
Reason for non-eligibility	**127**
Contraindication for corticosteroids	**98 (77.2%)**
Infection	**35 (27.5%)**
Renal disfunction	**26 (20.4%)**
Bleeding	**24 (18.8%)**
Pancreatitis	**9 (7.1%)**
Diabetes	**4 (3.1%)**
Others	**29 (22.8%)**
Maddrey > 90/MELD * > 51	**14 (11%)**
Rapid fatal evolution	**4 (3.1%)**
Unknown	**11 (8.6%)**

* MELD—Model for End-stage Liver Disease.

**Table 3:** Response rate and short- and long-term mortality of patients.

Responders, *n* (%)	138 (75.4%)
Non-responders, *n* (%)	45 (24.6%)
1-month mortality, *n* (%)	
Overall	38 (12.2%)
Corticosteroid responders	5 (3.6%)
Corticosteroid non-responders	16 (35.5%)
No corticosteroids	17 (13.3%)
1-year mortality, *n* (%)	
Overall	194 (62.5%)
Corticosteroid responders	59 (42.7%)
Corticosteroid non-responders	35 (77.7%)
No corticosteroids	100 (78.7%)

**Table 4:** Mortality risk according to corticosteroid administration and response.

Variable		Odds Ratio	95% CI		*p*-Value
			Lower	Upper	
1-month mortality	Responders	Reference			
	Non-responders	14.67	4.97	43.28	<0.001
	No corticosteroids	4.11	1.46	11.49	0.007
1-year mortality	Responders	Reference			
	Non-responders	4.68	2.15	10.21	<0.001
	No corticosteroids	4.95	2.88	8.53	<0.001

**Table 5:** Predictive factors for non-response to corticosteroids.

	Responders *n* = 138	Non-Responders *n* = 45	OR (CI 95%)	*p*-Value
**Age, years ^a^**	47 (38 to 54)	49 (45 to 63)	**1.05** (1.016–1.084)	**0.003**
Women, *n* (%) ^b^	50 (36%)	17 (38%)	0.93 (0.46–1.87)	0.85
**Preexistent cirrhosis, *n* (%)** ^b^	95 (69%)	23 (51%)	**2.11** (1.064–4.20)	**0.033**
Overt hepatic encephalopathy, *n* (%) ^b^	58 (42%)	28 (62%)	0.44 (0.22–0.88)	0.20
Ascites, *n* (%) ^b^	93 (67%)	37 (82%)	0.48 (0.19–1.03)	0.06
**Maddrey score ^a^**	57.1 (42.75 to 79.15)	74 (48.5 to 93.75)	**1.01** (1.01–1.03)	**0.015**
MELD score **^a^**	27 (24 to 31)	29 (25.5 to 34.5)	1.06 (0.99–1.12)	0.059
**MELD score > 30, *n* (%)** ^b^	47 (34%)	25 (56%)	**2.42** (1.21–4.8)	**0.011**
Platelets, × 10^9^/L **^a^**	135.5 (77.75 to 188.5)	110 (64.5 to 190)	1.09 (0.99–1.04)	0.65
Hemoglobin value **^a^**	11.7 (10.3 to 12.5)	10.6 (9.45 to 12.1)	0.91 (0.78–1.05)	0.20
Prothrombin time, s **^a^**	21.5 (18.2 to 25.6)	23.2 (17.85 to 26.9)	1 (0.97–1.04)	0.64
INR **^a^**	1.97 (1.7 to 2.35)	2.15 (1.69 to 2.55)	1.22 (0.82–1.82)	0.31
**Serum albumin, mg/dL ^a^**	2.67 (2.33 to 3.05)	2.45 (2.17 to 2.53)	0.42 (0.2–0.87)	0.02
**Severe hypoalbuminemia, (<2.8 g/dL)** ***n* (%)** ^b^	81 (59%)	35 (78%)	**2.46** (1.12–5.37)	**0.02**
Serum total bilirubin, mg/dL **^a^**	18 (12.15 to 26.95)	22.42 (15.75 to 28.5)	1.03 (0.99–1.07)	0.07
**Serum creatinine, mg/dL ^a^**	0.7 (0.55 to 1.01)	0.95 (0.69 to 1.85)	**1.5** (1.10–2.03)	**0.009**
Serum sodium, mmol/L **^a^**	133 (130 to 136)	132 (129 to 135)	0.97 (0.91–1.03)	0.41

Data expressed as ^a^ median [IQR] or ^b^ absolute (%).

## Data Availability

Data are contained within the article.
